# Introduction

**Published:** 1818

**Authors:** James Carver

**Affiliations:** V. S.


### INTRODUCTION.

It is only within these last thirty years that veterinary medicine in England has been placed on the footing of a science. The institution of the Royal Veterinary College of London is the great era from which this improvement may be dated, and the consequence of this has been, that ignorance and error have been detected, so as to establish what may be termed the difference between random routine and scientific practice; and the first professor, M. St. Bell, a Frenchman, showed (as I may be doing for this country) what now might be done during the short time he possessed the appointment; but his predecessor, professor Coleman, proceeded with more enlarged views on the subject, and with a mind ardent for improvement, he set out with instituting a close analogy between the diseases of man and the horse, and has now applied this analogy very happily to the *mutual illustration* of both, which only can be known by their different formation, and a comparison of the organs in the two different subjects.

Having in the body of this essay considered the best mode of treatment for one of the most fatal diseases that renders this animal of little or no value to his owner, I shall in the succeeding part of this work not only endeavour to impress on the mind of my readers how to learn the chief cause of the evil attendant on inflammation of the eyes of horses, but also *learn* them how to *prevent*, instead of *undertaking the tedious task of learning how to cure them*, in order that the present publication may fully answer the purpose for which it is intended.

The animal economy in its manifold relations is generally and fundamentally the same in man and beast, and governed by the same laws of nature and natural mechanics; but the greatest skill is requisite to form a judgment on the diseases of brutes, from their inability to describe their feelings, and the consequent uncertainty of their pathology: for can there be a greater burlesque, than the supposition of a man's ability to give physic for a horse, excepting in very common cases, merely because he knows how to groom and shoe him? The plea of experience is futile; for the utter inability of illiterate and uninformed men to investigate the principles of science, and their total want of opportunity to acquire, even by rote, a rational system of practice. The whole stock of medical knowledge o these practitioners usually consists of a number of receipts, handed down from Tom and Dick to Harry, with which they continually ring their changes in all cases, *right or wrong, hit or miss;* and so fiercely are they bigoted to their own particular nostrums, they are totally incapable of all advice or improvement, the common and unavoidable fate of confirmed ignorance, since it is the highest point of knowledge to know and feel that we still need information. Let not the reader suppose these to be mere flourishes; they are literal truths; and by the tenor of them he may judge of the majority of them throughout Europe, until the establishment of proper institutions with a view of drawing this useful and necessary branch of knowledge out of the hands of ignorant pretenders. Into such hands do men commit their distempered animals, who have it not in their power to reproach their masters with their accumulated sufferings. Mankind, from prejudice, indolence, and want of feeling, neglect those creatures which they can purchase with their money; and the progress of veterinary medicine, grounded, as it necessarily must be, on a proper knowledge of the anatomy, physiology, and pathology of disease, will in this country, as it was in Europe, be rapid or slow according to the diligence of those enlightened practitioners which the college has sent forth, to ascertain precisely the limits to which their knowledge extends, and in what cases it is defective.

Mr. Higgs, a very extensive practitioner of the present day in London, very justly observes, that as no labour can be done, or business carried on, in an extensive scale, but by the assistance of this useful animal, next to man he claims our first attention. In his natural state, he enjoys, with other animals, freedom from disease; but no sooner do we begin to domesticate him, do we entail disease upon him; and he is no longer the same animal as from the hand of Nature. This subject particularly interests both mercantile and commercial concerns, but which, in this country, I am sorry to add, has hitherto claimed a less share of attention than its importance deserves. My object, therefore, is to point out the best means of preserving him in health, by preventing disease; and in fact, all those minutiæ, from want of a little attention, to which his diseases chiefly arise, and, if possible, to invite and draw every proprietor to direct, in a certain degree, the care of an animal which constitutes to man many of the most valuable blessings of life.

A horse in town, and a horse in the country are, in some measure, not the same animal; and the observations which apply to the difference, which, as Mr. Higgs observes of a town and country life with man, applies equally here; and the number of horses annually lost in all the public cities of the United States, and particularly in the mail stage departments, either from evils attendant on bad shoeing or other mismanagement, is incalculable, and calls aloud for the attention and interference of government. They are of too much value to be so wilfully neglected or injured as they are; and there is no one, perhaps, but would follow proper directions, for his own interest, if he had them to go by. In endeavouring, therefore, to obviate this defect, I trust I am performing a most useful and acceptable task to the community, by the exertions I have, and still am making towards this desirable end; and if the opportunities which I have had in different parts of the world of acquiring a little more information than generally falls to the share of every veterinary practitioner, I shall die happy in the pleasing reflection that I have devoted those talents for the benefit of my adopted country.

JAS. CARVER, V. S.

